# Fecal gut microbiota and amino acids as noninvasive diagnostic biomarkers of Pediatric inflammatory bowel disease

**DOI:** 10.1080/19490976.2025.2517828

**Published:** 2025-06-12

**Authors:** Eva Vermeer, Jasmijn Z. Jagt, Eline M. Lap, Eduard A. Struys, Andries E. Budding, Nanda M. Verhoeven-Duif, Marjolein Bosma, Johan E. van Limbergen, Bart G.P. Koot, Robert de Jonge, Marc A. Benninga, Animesh Acharjee, Nanne K.H. de Boer, Tim G.J. de Meij

**Affiliations:** a Department of Paediatric Gastroenterology, Emma Children’s Hospital, Amsterdam UMC, Amsterdam, The Netherlands; b Amsterdam Gastroenterology Endocrinology Metabolism (AGEM) Research Institute, Amsterdam UMC, Amsterdam, The Netherlands; c Amsterdam Reproduction & Development (AR&D) Research Institute, Amsterdam UMC, Amsterdam, The Netherlands; d Faculty of Medicine, University of Amsterdam, Amsterdam, the Netherlands; e Department of Laboratory Medicine, Amsterdam UMC, Amsterdam, The Netherlands; f Inbiome BV, Amsterdam, The Netherlands; g Department of Genetics, Section Metabolic Diagnostics, UMC Utrecht, Utrecht, The Netherlands; h Institute of Cancer and Genomic Sciences, College of Medical and Dental Sciences, University of Birmingham, Birmingham, UK; i Centre for Health Data Research, University of Birmingham, Birmingham, UK; j Department of Gastroenterology and Hepatology, Amsterdam UMC, Amsterdam, The Netherlands

**Keywords:** Paediatric inflammatory bowel disease, gut microbiota, amino acids

## Abstract

**Background and Aims:**

Fecal calprotectin (FCP) has limited specificity as diagnostic biomarker of pediatric inflammatory bowel disease (IBD), leading to unnecessary invasive endoscopies. This study aimed to develop and validate a fecal microbiota and amino acid (AA)-based diagnostic model.

**Methods:**

Fecal samples from a discovery cohort (*de novo* IBD and healthy controls [HC]) were used to develop the diagnostic model. This model was applied in a validation cohort (*de novo* IBD and controls with gastrointestinal symptoms [CGI]). Microbiota and AAs were analyzed using interspace profiling and liquid chromatography-mass spectrometry techniques, respectively. Machine learning techniques were used to build the diagnostic model.

**Results:**

In the discovery cohort (58 IBD, 59 hC), two microbial species (*Escherichia coli* and *Alistipes finegoldii*) and four AAs (leucine, ornithine, taurine, and alpha-aminoadipic acid [AAD]) combined allowed for discrimination between both subgroups (AUC 0.94, 95% CI [0.89, 0.98]). In the validation cohort (43 IBD, 38 CGI), this panel of six markers could differentiate patients with IBD from CGI with an AUC of 0.84, 95% CI [0.67, 0.95]). Leucine showed the best diagnostic performance (AUC 0.89, 95% CI [0.81, 0.95]).

**Conclusions:**

Leucine might serve as adjuvant noninvasive biomarker in the diagnostic work-up of pediatric IBD. Future research should investigate whether the combination of leucine with FCP could improve specificity and may help tailor the course of diagnostics.

## Introduction

Inflammatory bowel disease (IBD) is characterized by chronic relapsing inflammation of the gastrointestinal tract, including Crohn’s disease (CD), ulcerative colitis (UC), and IBD-unclassified (IBD-U).^1^ Currently, 7–8% of IBD cases present before the age of 18 years.^2–4^ The diagnostic work-up of pediatric IBD relies on a combination of clinical symptoms along with laboratory, radiologic, endoscopic, and histologic findings.^5^ Faecal calprotectin (FCP) is the most widely used noninvasive diagnostic biomarker, which has a high sensitivity (98%) for detecting intestinal inflammation but a limited specificity (68%) in distinguishing between IBD and non-IBD controls.^6^ In children with elevated FCP levels, endoscopic assessment is presently required. However, this diagnostic approach is invasive, costly, and imposes a significant burden on children with a risk of complications.^7,8^ Consequently, there is a need to identify new noninvasive biomarkers with higher specificity, which could potentially improve the diagnostic approach to IBD.

Though the exact etiology of IBD remains largely unknown, the prevailing hypothesis is that it results from a complex interaction between host genetics, immune responses, environmental factors, and the gut microbiota and its related metabolites.^9^ Previous research has shown alterations in gut microbial composition between patients with IBD and healthy individuals in both children and adults, which mainly showed a decreased abundance of the phyla *Firmicutes* and *Bacteroidetes*, and increased abundance of members of the *Actinobacteria* and *Proteobacteria* phyla, especially *Enterobacteriaceae*, in IBD.^10–15^ Aside from the microbiota, the gut metabolome also influences intestinal immune homeostasis and mucosal integrity.^16^ Metabolomics holds the potential to unveil the complex interplay between the host, intestinal microbes, and environmental factors such as diet.^17,18^ Several studies have shown that fecal metabolomic profiles could differentiate between children with IBD and healthy controls (HC), with higher amino acid (AA) concentrations in pediatric IBD being the most consistent finding, primarily histidine, tryptophan, alanine, phenylalanine, taurine, leucine, tyrosine, and valine.^17,19,20^

Until now, the gut microbiome and metabolome have both separately shown their potential to accurately differentiate children with IBD from HC.^15,19,20^ This study aimed to develop a combined microbiota and AA-based model to discriminate between children with IBD and HC from a historical cohort.^15,20^ Subsequently, we aimed to prospectively validate this model in an independent cohort comprising children with IBD and controls with gastrointestinal symptoms (CGI).

## Materials and Methods

### Study design, patient population and sample collection in the discovery cohort

**Figure 1.**
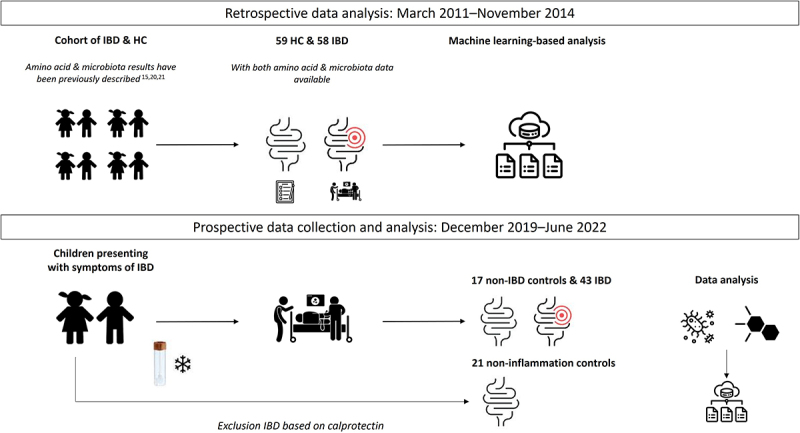
Flow chart of the discovery and validation cohort study design.

For the discovery cohort, fecal samples were selected from historical cohorts comprising children with newly diagnosed treatment-naïve IBD and HC, as illustrated in Figure 1. In these cohorts, fecal microbiota and AA profiles have been separately described.^15,20,21^ To develop a microbiota and AA-based diagnostic model, those children who had both their microbiota and the AA profiles analyzed from the same fecal sample were identified. The details of this discovery cohort, including fecal sample collection method and processing, are described in the Supplementary Methods and in previous studies.^15,20,21^

### Patient population and sample collection in the validation cohort

For validation of the developed model, based on the discovery cohort, a sample size of 80 participants (ratio 1:1 disease and controls) was calculated using the source web application PowerTools.^22^ A new independent validation cohort was used, which was embedded in a prospective longitudinal cohort study conducted at the Amsterdam University Medical Centres. The validation cohort comprised children with newly diagnosed treatment-naïve IBD and CGI, in whom IBD was excluded following diagnostic work-up based on current guidelines.^23,24^ The sample collection and storage were performed in a standardized manner using the same protocol as in the discovery cohort. Further details can be found in the Supplementary Methods.

### Microbiota analysis

The extraction of deoxyribonucleic acid (DNA) from prepared fecal samples was performed using the easyMAG kit (Biomérieux, Marcy l’Etoile, France) according to the manufacturer’s protocol.^25^ The fecal samples from both cohorts were analyzed by InterSpace Profiling (IS-pro), enabling differentiation between multiple bacteria based on the species-specific length of 16S-23S ribosomal DNA IS region.^25^ Details of DNA extraction and the IS-pro technique are described in the Supplementary Methods.

### Amino acid analysis

For AA analysis, fecal samples from both the discovery and validation cohort were prepared according to a previously described protocol.^20^ This protocol involved freeze-drying to reduce the risk of potential bias caused by differences in fecal water content. In the discovery cohort, targeted high-performance liquid chromatography (HPLC) was used to analyze AA concentrations in fecal samples from patients with IBD and HC. For the validation cohort, AA levels in fecal samples were analyzed using hydrophilic interaction liquid chromatography (HILIC) coupled to tandem mass-spectrometry (MS) at University Medical Centre Utrecht, The Netherlands.^26^ Details of the sample preparation and the liquid chromatography-mass spectrometry techniques can be found in the Supplementary Methods.

### Statistical analysis

The Statistical Package for Social Sciences (SPSS,IBM; v28) was used to perform statistical analyses on baseline characteristics for both cohorts. Means and standard deviations (SDs) were calculated for normally distributed data. For non-normally distributed data, medians and interquartile ranges (IQRs) were calculated. Demographic and clinical characteristics between groups were compared using the Mann-Whitney U test. For categorical variables, the χ^2^-test was applied. To investigate differences in baseline characteristics between children with IBD and controls from both cohorts, a p-value <0.05 was considered statistically significant.

### Machine learning-based analysis

An unsupervised principal component analysis (PCA) was used to explain variation across the discovery and validation datasets for both AA and microbiome data. Both data sets were normalized using autoscaling and subsequently applied in a supervised algorithm. Regularized methods including Least Absolute Shrinkage and Selection Operator (LASSO) and Elastic Net (EN) were used to identify the most stable features (AAs and microbes) classifying IBD versus HC.^27,28^ In each cohort, the samples were assigned either to a training or test set randomly. Ten-fold cross-validation was used to fine-tune the hyperparameters. Both EN and LASSO were used to choose features from the training set.

These techniques automate the identification of the most important features influencing the outcome variable (IBD versus non-IBD). LASSO penalizes the sum of absolute regression coefficients (L1), whereas EN uses a combination of L1 and squared values of the coefficients (L2) penalties. To rank the selected features, we performed 50 iterations. From these, the top 25% of features that were identified in both EN and LASSO were selected and used for subsequent logistic regression analysis, based on Bravo-Merodio et al.^29^ In this step, we tested multiple combinations of these selected features to determine the optimal combination that provided the best area under the receiver operating characteristic curve (ROC-AUC). The outcomes of the logistic regression analyses included the ROC-AUC, sensitivity, specificity, positive predictive value (PPV), negative predictive value (NPV), area under the precision – recall curve (PR-AUC), and F1-score.

## Results

### Patient characteristics

#### Discovery cohort

The participants in the discovery cohort, with both fecal microbiota and AA profiles available, consisted of 58 newly diagnosed children with IBD (28 CD and 30 UC) and 59 HC. Baseline characteristics are summarized in Table 1. Children with IBD had statistically significant differences in body mass index (BMI) (*p* < 0.05) and age (*p* < 0.001) when compared to HC (Mann-Whitney U tests).

**Table 1.** Descriptive characteristics of the discovery cohort and the validation cohort. Specific diagnoses of the control group of the validation cohort are depicted in supplementary tables.

	Discovery cohort				Validation cohort			
	IBD*	CD	UC	HC (n = 59)	IBD**	CD	UC	CGI
	(n = 58)	(n = 28)	(n = 30)		(n = 43)	(n = 24)	(n = 16)	(n = 38)
Age, years, median (IQR)	14 (11–15)	14 (13–15)	13 (9–15)	7 (5–11)	15.1 (11.1–16.4)	15.9 (12.1–16.9)	14.7 (10.6–15.4)	15.0 (12.9–16.6)
Males, n (%)	32 (55.2)	14 (50)	18 (60)	29 (49.2)	19 (44.2)	12 (50)	7 (43.8)	17 (44.7)
BMI, kg/m^2^, mean (SD)	18.1 (3.4)	17.8 (3.1)	18.3 (3.6)	16.6 (2.3)	19 (3.5)	19.5 (4.1)	18.2 (2.3)	19.2 (2.7)
Physician global assessment, n (%)				NA	NA	NA	NA	NA
Quiescent	0	0	0					
Mild	0	0	0					
Moderate	16 (27.6)	7 (25)	9 (30)					
Severe	41 (70.7)	21 (75)	20 (66.7)					
PCDAI baseline, median (IQR)	NA	NA	NA	NA	NA	32.5 (20–45)	NA	NA
PUCAI baseline, median (IQR)	NA	NA	NA	NA	NA	NA	45 (30–65)	NA
FCP, µg/mg, median (IQR)	1208 (552–1870)	1282 (711.8–1890)	648 (392–1840)	NA	2038 (1192.5–3416.8)	2050 (842–2860)	2237 (1401–5084)	41.5 (15.5–435.5)
CRP, mg/L, median (IQR)	10.0 (2.5–40)	33.0 (10–45)	2.5 (2.5–9.5)	NA	7.5 (1.0–23.8)	16.4 (6.0–53.3)	1.0 (0.7–5)	1.0 (0.3–2.7)
ESR, mm/H, median (IQR)	26.0 (15.3–41.3)	40.0 (19.0–50.0)	23.0 (8.0–35.0)	NA	18.5 (8–35.3)	32.5 (14.8–45.3)	12.0 (7.5–17.5)	8.0 (3.5–10.0)
Albumin, g/L, median (IQR)	37.0 (33.0–41.0)	36.0 (29.0–40.0)	37.5 (33.0–41.3)	NA	39 (35–43.5)	36 (30.7–39.8)	43.5 (39.2–45.3)	42 (39–47)
Paris location of patients with CD, n (%)	NA		NA	NA	NA		NA	NA
L1: ileal		3 (10.7)				6 (25)		
L2: colonic		9 (32.1)				4 (16.7)		
L3: ileocolonic		15 (53.6)				14 (58.3)		
L4a: Upper GI involvement		9 (32.1)				13 (54.2)		
L4b: Upper GI involvement		NA				6 (25)		
Paris behavior of patients with CD, n (%)	NA		NA	NA	NA		NA	NA
B1: non stricturing, nonpenetrating		16 (57.1)				22 (91.7)		
B2: stricturing		5 (17.9)				1 (4.2)		
B3: penetrating		6 (21.4)				1 (4.2)		
P: perianal disease, n(%)		4 (14.3)				4 (16.7)		
Paris location of patients with UC, n (%)	NA	NA		NA	NA	NA		NA
E1: ulcerative proctitis			2 (6.7)				2 (12.5)	
E2: left sided UC (distal splenic flexure)			6 (20)				7 (43.8)	
E3: extensive (hepatic flexure distally)			2 (6.7)				1 (6.3)	
E4: pancolitis (proximal hepatic flexure)			19 (63.3)				6 (37.5)	

*IBD group consists of 28 CD patients and 30 UC patients.

**IBD group consists of 24 CD patients, 16 UC patients and 3 IBD-U patients.

Abbreviations: CD, Crohn’s disease; UC, ulcerative colitis; IBD, inflammatory bowel disease; IBD-U, inflammatory bowel disease unclassified; BMI, body mass index; PCDAI, Paediatric Crohn’s Disease Activity Index; PUCAI, Paediatric Ulcerative Colitis Activity Index; FCP, fecal calprotectin; CRP, C-reactive protein; ESR, erythrocyte sedimentation rate; NA, non-applicable; HC, healthy controls; CGI, controls with gastrointestinal symptoms.

#### Validation cohort

The validation cohort consisted of 43 newly diagnosed children with IBD (24 CD, 16 UC and 3 IBD-U) and 38 CGI in whom IBD had been ruled out (Table 1). Seventeen out of 38 CGI (45%) underwent endoscopic examination to exclude IBD. The median FCP level in this subset of 17 CGI was 485 µg/mg (IQR 136–1042). In the other 21 CGI, IBD was ruled out following routine diagnostic work-up and based on clinical symptoms, laboratory findings including FCP and/or radiological results (median FCP was 20 µg/mg (IQR 10–70)). Approximately one year after these 21 GCI were included, electronic health records were reviewed to ensure that they did not develop IBD. The final diagnoses of CGI in the validation cohort are presented in Supplementary Table S1. Patients with IBD demonstrated significantly higher levels of FCP (*p* < 0.001), CRP (*p* < 0.001) and ESR (*p* < 0.001), and lower levels of albumin (*p* < 0.05) compared to the CGI. These p-values were derived using the Mann-Whitney U test. There were no statistically significant differences observed in age, gender or BMI within the validation cohort.

## Discovery cohort analysis

### Amino acid data analysis

In the discovery cohort, 42 unique AAs were identified and quantified using HPLC analysis. Of these AAs, nine were omitted due to missing values. The missing data were most likely caused by AA concentrations that were below the limit of detection, but they could also be due to co-eluting interferences preventing accurate quantification of the AA of interest. Therefore, we used 33 AAs to discover patterns in the data set. All the AA data were normalized using the auto-scaled method.

The variation explained by the AAs was assessed by PCA, showing distinct clustering between patients with IBD and HC. The AA profiles are also shown as a heat map presenting the up and down regulation of the AAs globally. To prioritize the AAs, we employed both LASSO and EN algorithms, using 10-fold cross-validation repeated 50 times. Each iteration selected key AAs. We then focused on the top 25th percentile of the features that appeared in both EN and LASSO, resulting in 18 selected AAs for downstream biomarker analysis. Further, we identified the optimal combination of selected AAs that provided the highest AUC values using logistic regression. This analysis revealed alpha-aminoadipic acid (AAD), ornithine, leucine, and taurine as the key AAs. Of these, ornithine, leucine, and taurine concentrations were significantly higher in fecal samples from patients with IBD versus HC in the discovery cohort (all showing p-values <0.001), whereas AAD did not show a significant difference. The performance of these combined AA features was analyzed through logistic regression analysis resulting in an ROC-AUC of 0.91 (95% CI [0.85–0.96]).

### Microbiome data analysis

The auto-scaled method was used to normalize the microbiome data in the discovery cohort. Sixty-nine unique interspace (IS) fragment lengths were included to investigate differences in abundance of microbiota species. The PCA using microbiota data demonstrated clustering of subjects within, and variation between, the group of patients with IBD and HC. We applied a similar methodology to that described in previous sections for the microbiome data. The top 25% of microbial features appearing in both EN and LASSO were *Escherichia coli* (nt 735–737 length position) and *Alistipes finegoldii* (nt 232). *E. coli* was upregulated in patients with IBD (*p* < 0.001), whereas *A. finegoldii* was downregulated compared with HC (*p* < 0.001). Logistic regression analysis of the combined microbial features resulted in an ROC-AUC of 0.87, 95% CI [0.81–0.95]).

### Integrative analysis of selected amino acid and microbiota markers

**Figure 2.**
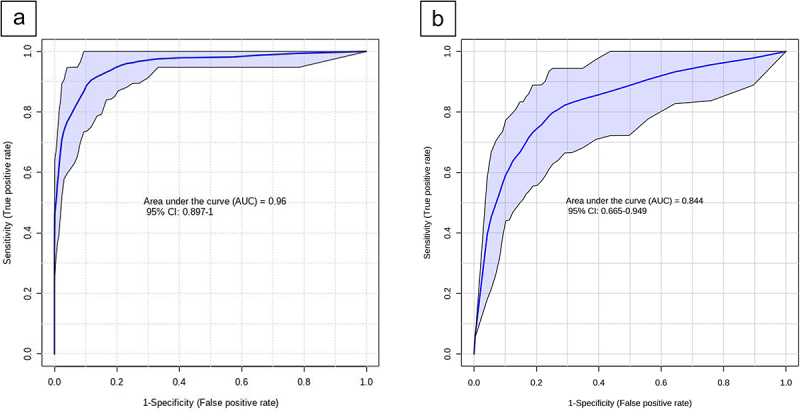
Receiver operating characteristic (ROC) curve with an area under the curve (AUC) related to the performance of the model using alpha-aminoadipic acid (AAD), ornithine, leucine, taurine, *E. coli*, and *A. finegoldii*. The light blue indicating the confidence interval of the estimates. (a) the AUC of the discovery cohort is 0.96, 95% CI [0.90, 1] (b) the AUC of the validation cohort is 0.84, 95% CI [0.67, 0.95].

An integrative analysis was performed, combining the selected features from the AA analysis (AAD, ornithine, leucine, and taurine) with those from the microbiota analysis (*E. coli* and *A. finegoldii*) in the discovery cohort. The highest accuracy of these six markers combined for differentiating IBD from HC was demonstrated using a logistic regression model, showing an ROC-AUC of 0.94 (95% CI [0.89, 0.98] based on a permutation test) (Figure 2(a)). The diagnostic performance of each individual marker is shown in Supplementary Figure S1, and the feature weights of the combined model can be found in Supplementary Table S2.

### Validation cohort analysis

**Figure 3.**
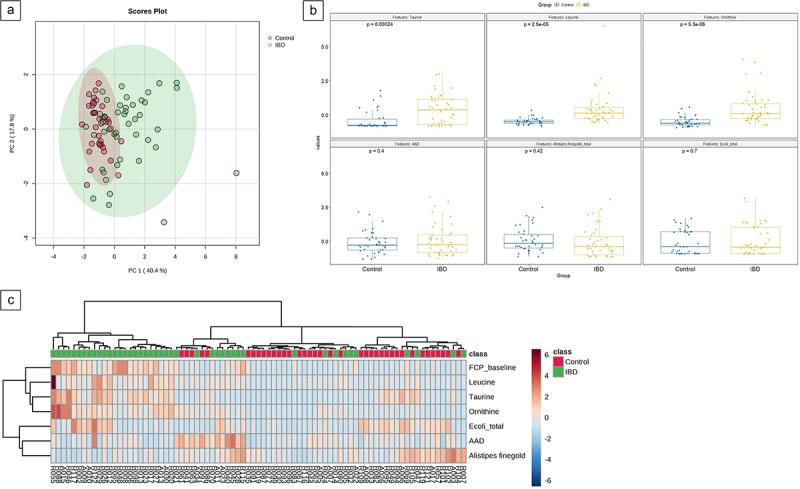
(a) Principal component analysis (PCA) score plot performed using six selected markers on children with IBD and controls with gastrointestinal symptoms (CGI), which shows clustering of subjects within, and variation between, groups. Dots represent samples and are colored according to the subject cohort. Ellipse represents 95% confidence. Results are plotted according to the PC1 and PC2 scores, with the percent variation explained by the respective axis. (b) box plots displaying the up and downregulated six markers in patients with IBD and CGI. A statistical significance noted from t-test at p-value (<0.05). (c) heat map visualizing selected markers within the validation cohort. The X-axis represents study participants classified into green (IBD) or red (controls), whereas the Y-axis represents different features. Blue and red colored blocks represent down- and upregulation of markers, respectively. Hierarchical clustering was applied to the rows (dendrograms) and similarly expressed markers were presented in the proximity.

The biomarker panel (2 microbiota and 4 AA markers) was subsequently validated on the independent cohort of children with IBD and CGI. A PCA was performed to explore variation and clustering within and between children with IBD and CGI (Figure 3(a)). Of the AA markers, ornithine, leucine, and taurine were significantly higher in children with IBD compared with CGI (all showing p-values <0.001), whereas AAD did not show a significant difference. The abundances of the microbial features *E. coli* and *A. finegoldii* were also not significantly different between IBD and CGI (Figure 3(b,c)).

**Figure 4.**
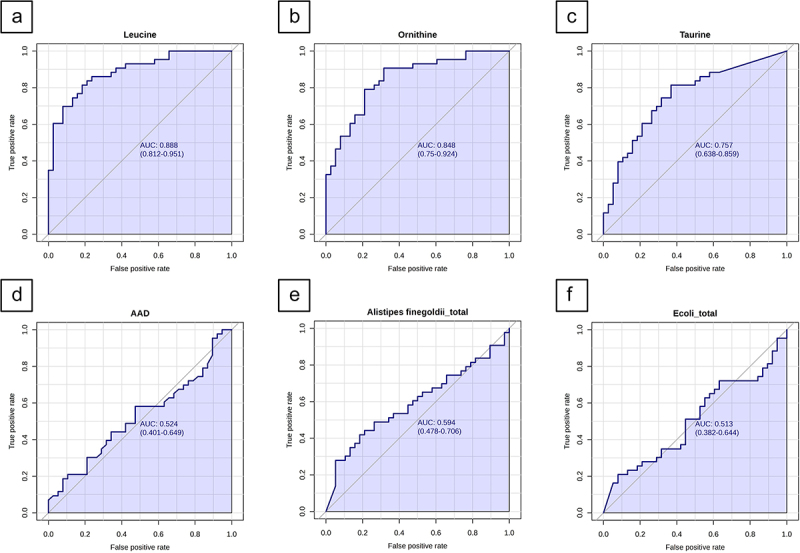
Receiver operating characteristic (ROC) curves of the six selected features within the validation cohort (a) leucine (b) ornithine (c) taurine (d) alpha-aminoadipic acid (e). *A. finegoldii* (f) *E. coli.*

The combined biomarker panel could differentiate children with IBD from CGI with an AUC of 0.84 (95% CI [0.67, 0.95]), sensitivity of 79%, and specificity of 87% (Figure 2(b)). When investigating these markers individually, leucine had the best diagnostic ability to differentiate between the subgroups (AUC = 0.89, 95% CI [0.81, 0.95], sensitivity 81%, specificity 81%, *p* < 0.001), as shown in Figure 4. This was followed by ornithine (AUC = 0.85, 95% CI [0.75, 0.92], *p* < 0.001), taurine (AUC = 0.74, 95% CI [0.64, 0.86], *p* < 0.001), *A. finegoldii* (AUC = 0.59, 95% CI [0.48, 0.71], *p* = 0.42), *E. coli* (AUC = 0.51, 95% CI [0.38, 0.64], *p* = 0.70), and AAD (AUC = 0.52, 95% CI [0.40, 0.65], *p* = 0.40). Figure 4 depicts the ROC-curves of the individual markers. Supplementary Table S3 presents the performance metrics of each individual biomarker as well as the combined model.

To assess the added value of our biomarker panel, we compared its diagnostic performance to models based on basic clinical features. A logistic regression model including age, sex, BMI, CRP, and FCP achieved an AUC of 0.91 (95% CI [0.85, 0.97]), with sensitivity of 83%, and specificity of 86%. A model using FCP alone performed similarly, with an AUC of 0.92 (95% CI [0.86, 0.98]), sensitivity of 86%, and specificity of 87%. Adding leucine to FCP further improved diagnostic performance, yielding an AUC of 0.95 (95% CI [0.92, 1.00]), with sensitivity of 88%, and specificity of 97%. Supplementary Table S4 depicts all performance metrics of the clinical models.

### Post-hoc analysis in a subgroup of IBD and CGI who underwent endoscopic examination

**Figure 5.**
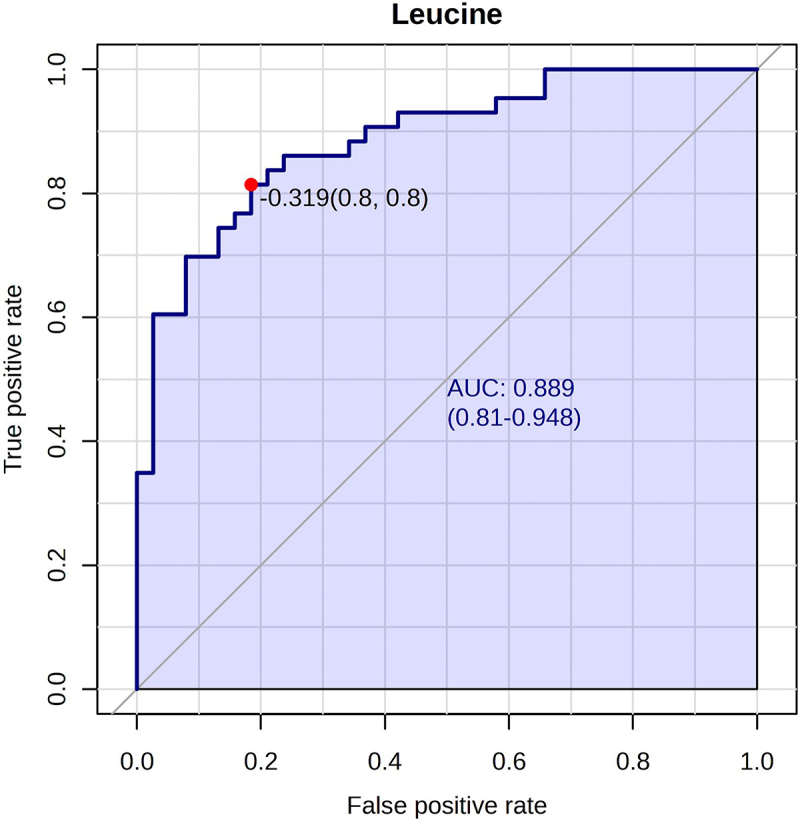
Receiver operating characteristic (ROC) curve with an area under the curve (AUC) related to the performance of the model in a subgroup of the validation cohort who underwent endoscopy, leucine. The AUC is 0.89, 95% CI [0.81, 0.95].

Since leucine showed the best performance to differentiate children with IBD from CGI, we subsequently investigated its diagnostic potential in a subgroup of the validation cohort consisting of all children with IBD (*n* = 43) and CGI who underwent endoscopic examination (*n* = 17). The median FCP level was 485 µg/mg (IQR 136–1042 µg/mg) and 2038 µg/mg (IQR 1193 – 3417 µg/mg) in the CGI and children with IBD, respectively. In this subgroup, leucine showed an ROC-AUC of 0.89 (95% CI [0.81, 0.95], *p* < 0.001) with a sensitivity of 81%, and a specificity of 81% (Figure 5).

Additionally, logistic regression defined an optimal cutoff value of leucine of 260 µM, yielding a sensitivity of 81% and a specificity of 81%.

## Discussion

This study aimed to develop and validate a microbiota and AA-based diagnostic model to differentiate between children with IBD and CGI. In the discovery cohort, a biomarker panel of two microbial species (*E. coli and A. finegoldii*) and four AAs (ornithine, leucine, taurine, and AAD) could differentiate children with IBD from HC with an AUC of 0.94. In the validation cohort, this panel showed an AUC of 0.84 for discriminating children with IBD from CGI. Leucine showed the best diagnostic performance (AUC 0.89), whereas the other five markers did not contribute to a better overall AUC.

Bosch et al. found increased levels of fecal leucine in children with IBD (*n* = 30) compared with HC (*n* = 15, AUC 0.82), along with an increase in other AAs including tryptophan, histidine, phenylalanine, tyrosine, and valine.^19^ In a study by the same research group, leucine was also significantly more abundant in the pediatric IBD group (*n* = 11) compared to CGI (*n* = 8, AUC 0.98).^30^ A common hypothesis is that increased levels of fecal AAs can be attributed to loss of AAs through increased permeability of the gut.^31^ Several studies have shown that a disruption of the mucosal barrier and thus increased permeability may occur early in IBD pathogenesis,^32,33^ and microbial dysbiosis in IBD may further contribute to and maintain the intestinal barrier dysfunction.^34^

Microbial analysis of the discovery cohort showed an increased abundance of *E. coli* and downregulation of *A. finegoldii* in the IBD group versus HC, which is consistent with the results of a large multi-omics study including both adults and children with IBD.^14^ In the validation cohort, these microbes did not differ significantly between children with IBD and CGI. A possible explanation for this apparent discrepancy between both cohorts could be the dissimilarity of the control groups. The discovery cohort included HC, whereas CGI were included in the validation cohort, of whom 66% were diagnosed with functional abdominal pain disorders, including irritable bowel syndrome (IBS) and functional abdominal pain-not otherwise specified. IBS in particular has been associated with dysbiosis of gut microbial composition in both children and adults, characterized by increased abundance of *Enterobacteriaceae* (of which *E. coli* is a member), *Lactobacillaceae*, and *Bacteroides*, decreased abundance of Clostridiales, *Faecalibacterium*, and *Bifidobacterium*, and decreased microbial diversity compared with healthy individuals.^35–38^ Specifically, fecal levels of *Alistipes spp*. have been reported to be lower in patients with IBS than HC, which could explain that *A. finegoldii* could not accurately discriminate between IBD and CGI.^39^ *Enterobacteriaceae*, and Proteobacteria in general have been associated with pro-inflammatory properties, while members of the Bacteroidetes phylum are known to have anti-inflammatory properties.^13,40,41^ Whether these changes in gut microbial composition are the underlying cause of IBD or a consequence of prolonged intestinal inflammation remains uncertain.^13,42^

In a post-hoc analysis of a subgroup of 60 patients from the validation cohort who underwent endoscopy, we found that leucine significantly distinguished children with IBD from controls, with an AUC of 0.89. This suggests that our prediction model performs well even in cases where it is challenging to diagnose IBD based on symptoms and conventional biomarkers, such as FCP, erythrocyte sedimentation rate (ESR), and albumin. If implemented, the model could reduce the need for unnecessary endoscopies in this group of patients. While the study design does not allow for definitive conclusions based on this post-hoc analysis, it offers promising directions for future research.

The strength of this study is the use of machine learning techniques to develop a fecal omics-based prediction model that could improve the specificity and sensitivity of pediatric IBD diagnostic models, supporting previous studies in adults with IBD.^43–45^ Secondly, an external validation cohort, using an orthogonal analytical method to measure fecal AAs, was included to apply the prediction model, showing the potential of leucine as an adjuvant diagnostic biomarker of pediatric IBD. Only newly diagnosed, treatment-naïve children were included, minimizing the risk of bias caused by IBD medication use. All fecal samples were collected in a standardized manner in the week before bowel preparation, avoiding the influence of laxatives on the gut microbial composition.^46^

This study also has several limitations. Although the age difference between HC and IBD patients in the discovery cohort was statistically significant, it is unlikely to have impacted the outcome, as the intestinal microbiome stabilizes and attains an adult-like composition by approximately three years of age.^47,48^ In addition, data on environmental factors such as diet were not assessed in a standardized manner, which could have affected the observed differences in microbial and AA profiles. Moreover, the control group in the validation cohort consisted of both non-IBD inflammation subjects and non-inflammation subjects. Previous studies have found that some bacterial species have pro-inflammatory or anti-inflammatory properties, which could mean that there is a difference in microbial and metabolomic profiles between inflamed and non-inflamed intestinal mucosa.^13,40,41^ Fifty-five percent of the CGI in the validation cohort did not undergo an endoscopic procedure as part of the diagnostic workup. In these cases, IBD was ruled out based on low FCP (median 20 µg/mg) and other inflammatory markers such as BSE and albumin. However, at one-year follow up, none of these individuals had received an IBD diagnosis. Since only one sample per patient has been collected and analyzed, we were not able to assess the intra-individual variability of the microbiota and AA profiles over time. Future prospective longitudinal studies should investigate this intra-individual variability of leucine and its stability under different preservation conditions.

The main goal of our study was to develop a microbiome- and metabolome-based diagnostic model that improves upon existing tools, such as FCP and clinical variables, which often show suboptimal specificity in routine care. While FCP is known for its high sensitivity (95%), its specificity is relatively low (68%), leading to potential over-referral and avoidable endoscopies.^6^ Our findings demonstrate that the addition of leucine to FCP markedly increases diagnostic specificity (97%). A combined test of leucine and FCP could be relatively easy to implement in clinical practice. Leucine is measured through LC-MS, which is increasingly used for clinical applications in laboratory medicine.^49^ In our validation cohort, FCP alone misclassified 19 out of 38 CGI as false positives, resulting in a specificity of just 50%.

Seventeen CGI underwent endoscopic examination, of whom 14 had increased FCP levels. Of those 14 CGI with elevated FCP levels, only five patients also showed high leucine concentrations, based on an optimal cutoff of 260 µM (sensitivity 81%, specificity 81%). Hence, it could be speculated that adding leucine to FCP could have prevented nine endoscopic procedures. Future prospective, multi-center and preferably multi-country studies are recommended to evaluate the performance of a combined leucine with FCP test versus FCP alone in the diagnostic work-up of pediatric IBD. This would assess the reproducibility and generalizability of leucine as a diagnostic marker across a diverse patient population, including children from multiple ethnic backgrounds exposed to different environmental factors and dietary patterns. To investigate whether leucine also holds the potential to serve as a biomarker for disease monitoring, prediction of treatment response, or even as a therapeutic target, future longitudinal research is warranted.

## Conclusions

Leucine seems to hold promise as an adjuvant, noninvasive diagnostic biomarker for children presenting with symptoms of IBD. The combination of leucine and FCP might improve the diagnostic potential of noninvasive fecal tests and is therefore recommended to be the subject of future research. This could contribute to a reduction of the number of unnecessary referrals for endoscopic examinations.

## Supplementary Material

Supplementary Materials_v3_Gut Microbes.docx

## Data Availability

The anonymized and coded data underlying this article have been uploaded to a publicly accessible repository, which can be accessed by visiting 10.6084/m9.figshare.28749422.

## Manuscript approval

EV, JJ, EL, ES, AB, NVD, MB, JvL, BK, RdJ, MAB, AA, NdB, and TdM declare that they have seen and approved the final version of the manuscript.

## Originality statement

The manuscript, including related data, figures and tables has not been previously published and the manuscript is not under consideration elsewhere.

## Abbreviations

AA: amino acid
AAD: alpha-aminoadipic acid
AUC: area under the curve
BMI: body mass index
CD: Crohn’s disease
CGI: controls with gastrointestinal symptoms
CRP: C-reactive protein
EN: Elastic Net
ESR: erythrocyte sedimentation rate
FCP: fecal calprotectin
HC: healthy controls
HILIC: hydrophilic interaction liquid chromatography
HPLC: high-performance liquid chromatography
IBD: inflammatory bowel disease
IBD-U: inflammatory bowel disease-unclassified
IBS: irritable bowel syndrome
IS-Pro: InterSpace Profiling
LASSO: Least Absolute Shrinkage and Selection Operator
LC-MS: liquid chromatography-mass spectrometry
MS: mass spectrometry
NPV: negative predictive value
PCA: principal component analysis
PCDAI: Paediatric Crohn’s Disease Activity Index
PGA: physician global assessment
PPV: positive predictive value
PUCAI: Paediatric Ulcerative Colitis Activity Index
UC: ulcerative colitis
