# Supplementary material for: Fecal gut microbiota and amino acids as noninvasive diagnostic biomarkers of Pediatric inflammatory bowel disease
Source: Gut Microbes. 2025 Jun 12;17(1):2517828. doi: 10.1080/19490976.2025.2517828 (PMC12164387; doi:10.1080/19490976.2025.2517828)
Supplement: Supplementary Materials_v3_Gut Microbes.docx [file KGMI_A_2517828_SM1409.docx]

**Supplementary Methods**

**Details of study design, patient population and sample collection in the discovery cohort**

The discovery cohort consisted of children (< 18 years of age) with treatment-naïve IBD, who were included between March 2011 and November 2014 at Amsterdam University Medical Centres, locations Academic Medical Centre (AMC) and VU Medical Centre (VUMC), the Netherlands. In summary, children diagnosed with CD or UC according to the revised Porto criteria were eligible to participate, whereas patients with IBD-U were excluded^5^. Disease localisation and behaviour were classified based on the Paris classification. Exclusion criteria were: [1] proven infectious colitis within three months prior to inclusion; and [2] use of antibiotics, steroids, or immunosuppressive therapy within one month prior to inclusion. Biomarkers including C-reactive protein (CRP), erythrocyte sedimentation rate (ESR), FCP, and albumin were evaluated at time of diagnosis. Disease activity was assessed according to the physician global assessment (PGA) score. Participants collected faecal samples at home prior to bowel cleansing using an instruction form. Samples were transported from participants’ freezers at home to the hospital for storage at -20°C until further processing occurred. Faecal samples from 59 HC from the same region in the Netherlands, without gastrointestinal symptoms, served as the control group. The details of this control group have been previously described. Briefly, healthy children from primary and secondary schools across five regions of the Netherlands were recruited to provide faecal samples. The same exclusion criteria and study protocol for sample collection and storage were applied as in the IBD group. Follow-up occurred 18 months after inclusion, during which children and/or their parents reported any major health changes, including the development of chronic or transient gastrointestinal diseases.

This study was approved by the Medical Ethical Review Committee of the Amsterdam University Medical Centres under file number 2015.393. Verbal consent was obtained from the parents or guardians and from the participating children.

**Details of patient population and sample collection in the validation cohort**

Patients aged 4–17 years referred to the outpatient clinic of the Amsterdam University Medical Centres between December 2019 and June 2022 with gastrointestinal symptoms that required diagnostic work-up for IBD, were eligible for inclusion. Exclusion criteria were [1] an immunocompromised disease; [2] the use of antibiotics or probiotics within three months prior to inclusion; and [3] the use of immunosuppressive medication. Patients were allocated to the IBD or CGI group following diagnostic work-up according to current guidelines^23,24^. Disease phenotype was classified according to the Paris classification. Paediatric Ulcerative Colitis Activity Index (PUCAI) or Paediatric Crohn’s Disease Activity Index (PCDAI) were used to assess disease activity. The CGI consisted of controls in which IBD was excluded based on FCP and controls who required endoscopic examination to rule out IBD. The following data was collected from all participants: demographic data such as age, gender, and body mass index (BMI), and levels of biomarkers including CRP, ESR, FCP, and albumin.

Patients were requested to collect a faecal sample prior to bowel cleansing for endoscopic assessment and to store these in a provided container in the household freezer directly after collection. For CGI, stool samples were collected at the time of inclusion. Faecal samples were transported under cooled conditions to the hospital within one week after collection and were consequently stored at -80°C.

The Medical Ethical Review Committee of the Amsterdam University Medical Centres has ethically reviewed and approved this validation study (file number 2020.362). Written informed consent has been obtained from children and their parents or guardians.

**Microbiota analysis**

**Sample preparation and DNA extraction**

A 100-400 mg aliquot of faecal sample was mixed with 200 μL of nucliSENS lysis buffer in an Eppendorf tube and subsequently vortexed for 5 minutes. This mixture was centrifuged at 13000 rpm for 2 minutes. Following centrifugation, 100 μL of the supernatant was transferred to an easyMAG isolation container containing 2 mL of nucliSENS lysis buffer. Seventy μL of magnetic silica beads were added after incubation at room temperature for 10 minutes. According to the protocol, deoxyribonucleic acid (DNA) was eluted in 110 μL buffer using the easyMAG automated DNA isolation machine (Biomérieux, Marcy l'Etoile, France)^25^.

**InterSpace Profiling for microbiota analysis**

InterSpace Profiling (IS-pro) was used for microbiota analysis. This technique enables differentiation between multiple bacteria in the samples using the species-specific length of 16S-23S ribosomal DNA IS region. The measured length of this IS region, expressed as a number of nucleotides, identified bacteria at species level using a database consisting of > 1500 species and their corresponding IS lengths. Phylum-specific colour labelling with fluorescent primers provides profiles which differentiate bacteria at the phylum and species level^25^. These profiles yield information on relative abundance of species since peak height reflects quantity. The IS-pro assay (IS-diagnostic Ltd, Amsterdam, the Netherlands) protocol was followed to amplify IS-regions^25^.

**Amino acid analysis
Sample preparation**
In both cohorts, a 300 mg aliquot per faecal sample was mixed with 1000 μL of distilled water by vortex for one minute. To reduce risk of potential bias caused by differences in faecal water content, samples were freeze-dried for at least 6 and maximum 24 hours (Crist Alpha 2-4). The remaining 30-50 mg of the sample was blended by vortex with distilled water with a faeces-water ratio of 100 mg : 5 mL. Of this mixture, 400 μL was pipetted into a filter and centrifuged at 14,000 g for 20 minutes (Hettig Zentrifugen Mikro 2R).

**Amino acid analysis method in discovery cohort**

Targeted high-performance liquid chromatography (HPLC) was used to analyse AA concentrations in faecal samples from patients with IBD and HC. An internal standard solution was mixed with the supernatant with a 1:1 ratio. The resulting mixture was centrifuged for 10 minutes (Whatman, Buckinghamshire, UK), after which the mixture was filtered into containers for final AA analysis (Biochrome 30). Ion-exchange chromatography was used to separate AAs. Detection of AAs occurred with UV absorbance after derivatisation with ninhydrin.

**Amino acid analysis method in validation cohort**

AA concentrations in prepared faecal samples of the validation cohort were analysed using hydrophilic interaction liquid chromatography (HILIC) coupled to tandem mass-spectrometry (MS) at University Medical Centre Utrecht, The Netherlands**.** Faecal samples were mixed with an internal standard solution maintaining a 1:1 ratio and mixed by vortex. The used AA mixture consisted of alpha-aminoadipic acid (AAD), ornithine, leucine, and taurine. After vortexing, a solvent (10 nM ammoniumformiate in 85% acetonitrile + 0.15% formic acid) was added with a sample-solvent ratio of 1:7 and mixed by vortex again. This mixture was centrifuged in an Eppendorf centrifuge at 17,000 xg for 5 minutes. The supernatant was consequently transferred to a 96 wells-plate (Waters, Etten-leur). The samples were filtered with a 0.2 μM GHP-filter (VWR, Amsterdam). After filtration, chromatographic separation of AAs was performed on a Acquity UPLC BEH Amide column including a Van Guard^TM^ UPLC BEH Amide pre-column (Waters, Milford, USA). The column was coupled to a Xevo-TQ MS triple quadrupole mass spectrometer with an electrospray ionisation (ESI) source and an Acquity UPLC-system (Waters, Manchester, United Kingdom). Details regarding analysis and used methods have extensively been described in previous research^26^.

**Supplementary Tables**

**Supplementary Table S1.** Final diagnosis of controls of the validation cohort and number of performed endoscopies for each category.

| **Final diagnosis** | **Patients, n (%)** | **Endoscopy, n (%)** |
| --- | --- | --- |
| H. pylori gastritis/duodenitis | 1 (2.6) | 1 (100) |
| Functional gastrointestinal disorders (irritable bowel syndrome/functional abdominal pain/functional constipation) | 25 (65.8) | 5 (21.7) |
| Infectious colitis | 3 (7.9) | 2 (66.7) |
| Polyp | 4 (10.5) | 4 (100) |
| Aspecific colitis | 1 (2.6) | 1 (100) |
| Complication after previous surgery | 1 (2.6) | 1 (100) |
| Coeliac disease | 1 (2.6) | 1 (100) |
| Protein losing enteropathy and panniculitis | 1 (2.6) | 1 (100) |
| Chronic appendicitis | 1 (2.6) | 1 (100) |

**Supplementary Table S2.** Feature weights of the combined models in both the discovery cohort and the validation cohort.

|  | **Discovery cohort** | | **Validation cohort** | |
| --- | --- | --- | --- | --- |
|  | **Abs (Estimate)** | **Std. Error** | **Abs (Estimate)** | **Std. Error** |
| **Taurine** | 4.417 | 2.427 | 0.154 | 0.499 |
| **Leucine** | 0.066 | 1.484 | 4.328 | 1.471 |
| **Ornithine** | 4.989 | 2.583 | 1.256 | 0.862 |
| **AAD** | 3.21 | 1.056 | 0.226 | 0.368 |
| ***Alistipes finegoldii*** | 1.302 | 0.567 | 0.473 | 0.375 |
| ***Escherichia coli*** | 1.605 | 0.461 | 0.495 | 0.4 |

**Supplementary Table S3.** Performance metrics of each individual biomarker as well as the combined model, including all six biomarkers. NPV could not be estimated for *E. coli* due to class imbalance, with insufficient true negatives available.

Abbreviations: ROC-AUC, area under the receiver operating characteristic curve; 95% CI, 95% confidence interval; PPV, positive predictive value; NPV, negative predictive value; PR-AUC, area under the precision–recall curve; AAD, amino-adipic acid; NA, not available.

|  | **ROC-AUC**  **(95% CI)** | **Sensitivity**  **(%)** | **Specificity**  **(%)** | **PPV**  **(%)** | **NPV**  **(%)** | **PR-AUC**  **(95% CI)** | **F1-score**  **(95% CI)** | **P-value** |
| --- | --- | --- | --- | --- | --- | --- | --- | --- |
| **Combined biomarker panel** | 0.84  (0.67-0.95) | 79.1 | 86.8 | 87.2 | 78.6 | 0.88  (0.60-0.94) | 0.79  (0.63-0.95) | NA |
| **Leucine** | 0.89  (0.81-0.95) | 81.3 | 81.4 | 83.4 | 79.4 | 0.91  (0.81-0.98) | 0.80  (0.65-0.90) | < 0.001 |
| **Ornithine** | 0.85  (0.75-0.92) | 72.1 | 78.9 | 79.5 | 71.4 | 0.87  (0.74-0.96) | 0.75  (0.57-0.86) | < 0.001 |
| **Taurine** | 0.74  (0.64-0.86) | 60.5 | 78.9 | 76.5 | 63.8 | 0.77  (0.59-0.92) | 0.64  (0.47-0.78) | < 0.001 |
| **AAD** | 0.52  (0.40-0.65) | 69.8 | 26.3 | 51.7 | 43.5 | 0.56  (0.31-0.76) | 0.52  (0.26-0.73) | 0.40 |
| ***Alistipes finegoldii*** | 0.59  (0.48-0.71) | 79.1 | 21.1 | 53.1 | 47.1 | 0.63  (0.36-0.80) | 0.59  (0.29-0.74) | 0.42 |
| ***Escherichia coli*** | 0.51  (0.38-0.64) | 1.0 | 0.0 | 53.1 | NA | 0.55  (0.35-0.69) | 0.52  (0.20-0.75) | 0.70 |

**Supplementary Table S4.** Performance metrics of models based on clinical features. The combined clinical model includes age, sex, BMI, CRP, and FCP. The combined model of clinical features and biomarker panel includes leucine, ornithine, taurine, AAD, *A. finegoldii*, *E. coli*, age, sex, BMI, CRP, and FCP.

Abbreviations: ROC-AUC, area under the receiver operating characteristic curve; 95% CI, 95% confidence interval; PPV, positive predictive value; NPV, negative predictive value; PR-AUC, area under the precision–recall curve; FCP, faecal calprotectin; BMI, body mass index; CRP, C-reactive protein; AAD, amino-adipic acid.

|  | **ROC-AUC**  **(95% CI)** | **Sensitivity**  **(%)** | **Specificity**  **(%)** | **PPV**  **(%)** | **NPV**  **(%)** | **PR-AUC**  **(95% CI)** | **F1-score**  **(95% CI)** |
| --- | --- | --- | --- | --- | --- | --- | --- |
| **FCP** | 0.92  (0.86-0.98) | 86 | 87 | 88 | 84 | 0.94  (0.86-0.99) | 0.84  (0.69-0.96) |
| **Combined clinical model** | 0.91  (0.85-0.97) | 83 | 86 | 88 | 75 | 0.95  (0.71-1.0) | 0.82  (0.65-0.95) |
| **Combined model clinical features + biomarker panel** | 0.91  (0.69-0.97) | 88 | 86 | 82 | 75 | 0.82  (0.63-0.95) | 0.83  (0.64-0.99) |
| **FCP + leucine** | 0.95  (0.92-1.00) | 88 | 97 | 97 | 88 | 0.96  (0.90-1.0) | 0.90  (0.80-1.0) |

**Supplementary Figures**

**Supplementary Figure S1.** Receiver operating characteristic (ROC) curves of the six selected features within the discovery cohort **A.** *A. finegoldii* **B.** *E. coli*

**C.** Leucine **D.** Ornithine **E.** Taurine **F.** Alpha-aminoadipic acid.

***
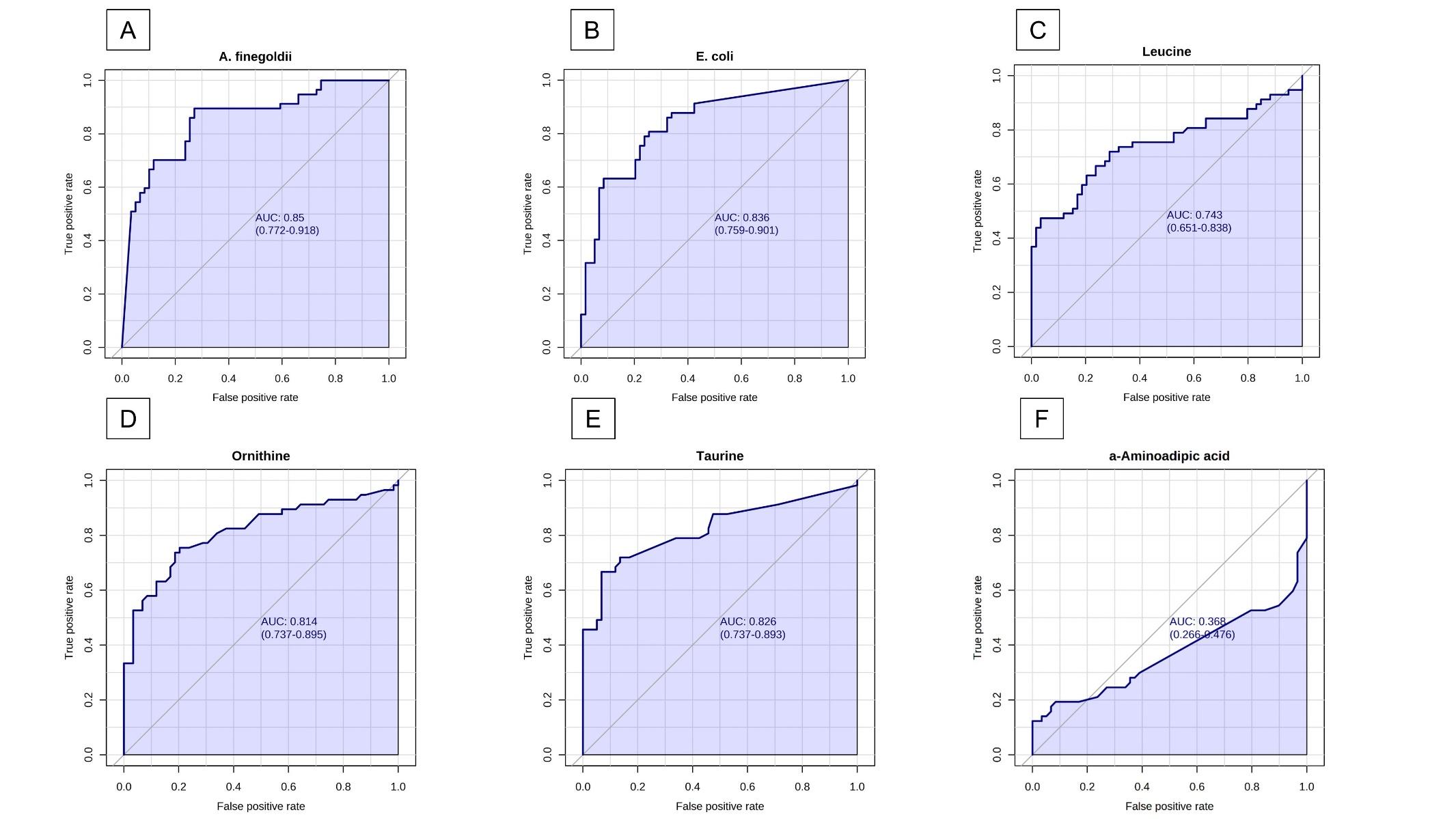
***
